# Photographic smile tracking: Evidence of asymmetric crying faces over time improvement: Case report

**DOI:** 10.1016/j.ijscr.2020.05.042

**Published:** 2020-05-29

**Authors:** Maamouri Sabrine, Marouen Ben Rejeb, Karima Zitouni, Issam Zairi

**Affiliations:** Department of Oral, Maxillofacial and Cosmetic Surgery, University Hospital of Charles Nicolle, 1006, Tunis, Tunisia

**Keywords:** Asymmetric crying faces, Smile, Nerve palsy

## Abstract

**Introduction:**

Congenital hypoplasia of the depressor anguli oris muscle is a rare mimic disorder depicted by a lower lip asymmetry apparent when laughing or crying.

**Presentation of case:**

A 10-year-old boy consulted our department for an asymmetry when opening his mouth. According to the family, this asymmetry was present since birth. Perinatal characteristics and childhood medical history were investigated with no abnormalities.

Physical exam revealed an inability to draw down the right lower lip unilaterally. At rest position, facial asymmetry was not noticeable.

Several investigations were done: A CT scan of the petrous part of the temporal bone, an MRI of soft tissues, an electromyography and a heart ultrasound. No anomalies were found.

**Discussion:**

This condition has stimulated great interest because of its potential association with congenital anomalies but also in order to reassure families often worried by the situation.

A large therapeutic armamentarium is described in literature.

**Conclusion:**

Among the large armamentarium of therapeutic options, we opted for a wait and see strategy through photographic smile tracking leading to an evidence of Asymmetric crying faces over time improvement.

## Highlights

• Congenital hypoplasia of the depressor anguli oris muscle is a rare mimic disorder.
• Drawing a distinction with a facial nerve palsy.
• Diagnosing the potential associated congenital anomalies specially the cardiac anomalies.
• There is a large armamentarium of therapeutic options.
• Proposing a wait and see approach since this condition improves through time.

## 1 Introduction

Congenital hypoplasia of the depressor anguli oris muscle (CHDAOM) [1]; “Asymmetric crying faces” [2]; or “Congenital lower lip palsy” (CULLP) as stated by Kobayashi [3] are different terminologies used to characterize a mimic disorder depicted by a lower lip asymmetry similar to a palsy of the marginal mandibular branch apparent when laughing, crying or “showing teeth”.

The importance of recognizing this rare entity lies in the fact that there is strong association of this condition with other significant anomalies. If it’s presented as an isolated anomaly, no treatment is required because the asymmetry is not noticeable in a grown-up child.

This work has been reported in line with the SCARE criteria [4].

## 2 Case report

A 10-year-old boy consulted our department for an asymmetry when opening his mouth. According to the family, this asymmetry was present since birth. Perinatal characteristics and childhood medical history were also investigated with no abnormalities: no consanguinity, a well monitored pregnancy without issues, vaginal delivery without instrumental assistance at full term.

Physical exam revealed an inability to draw down the right lower lip unilaterally. At rest position, facial asymmetry was not noticeable, but became evident particularly when opening the mouth and laughing. Other mimetic movements such as forehead wrinkling, eye closure and pouting were normal. No functional disorders in pronunciation were noticed.

Several investigations were done; A CT scan of the petrous part of the temporal bone and MRI of facial soft tissues, an electromyography and a heart ultrasound: no anomalies were found

We adopted a “wait and see strategy “and tracked the evolution of this mimic disorder through 10 years: one picture per year like in the annexed figures (Fig. 1), (Fig. 2), (Fig. 3), (Fig. 4) (Fig. 5), (Fig. 6), (Fig. 7), (Fig. 8), (Fig. 9), (Fig. 10).

**Fig. 1:**
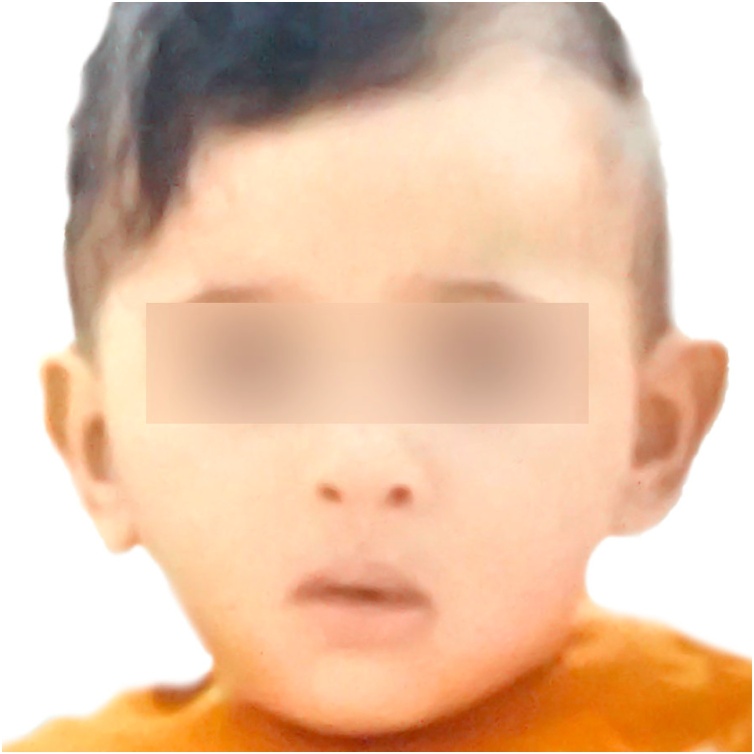
1 year old.

**Fig. 2:**
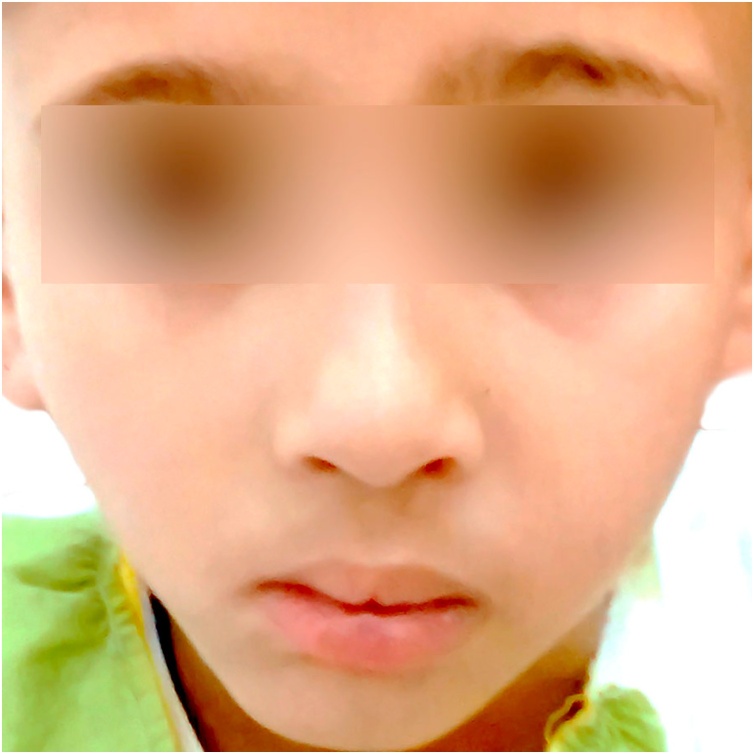
2 years old.

**Fig. 3:**
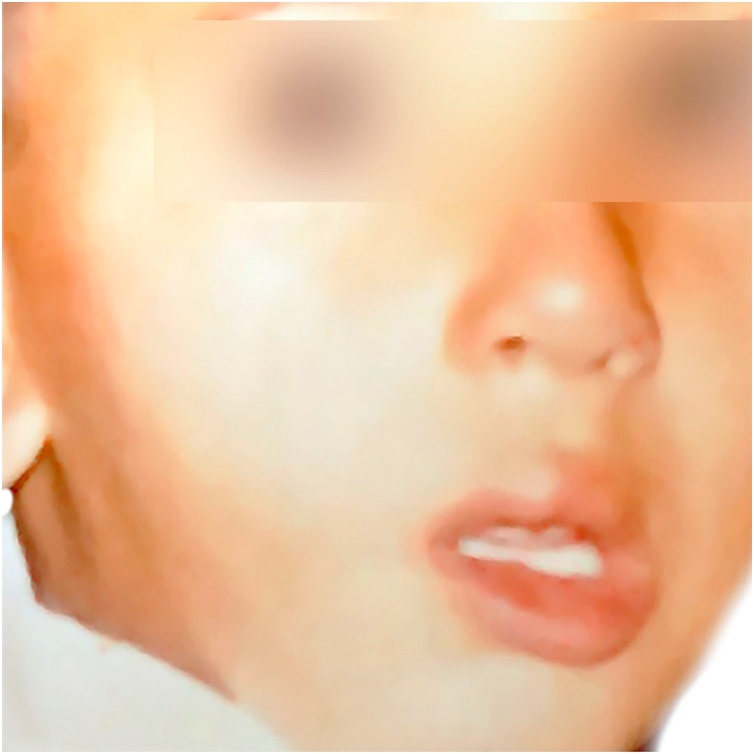
3 years old.

**Fig. 4:**
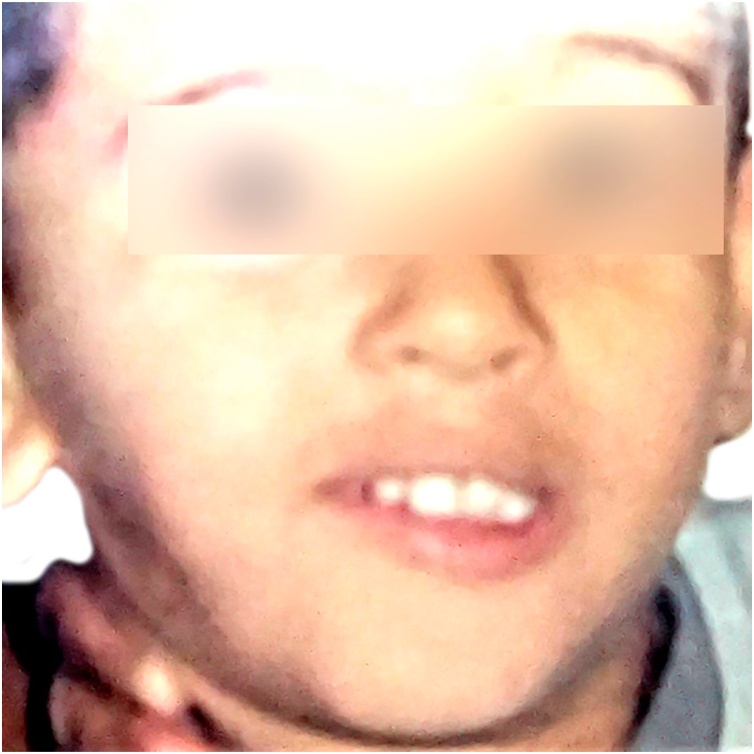
4 years old.

**Fig. 5:**
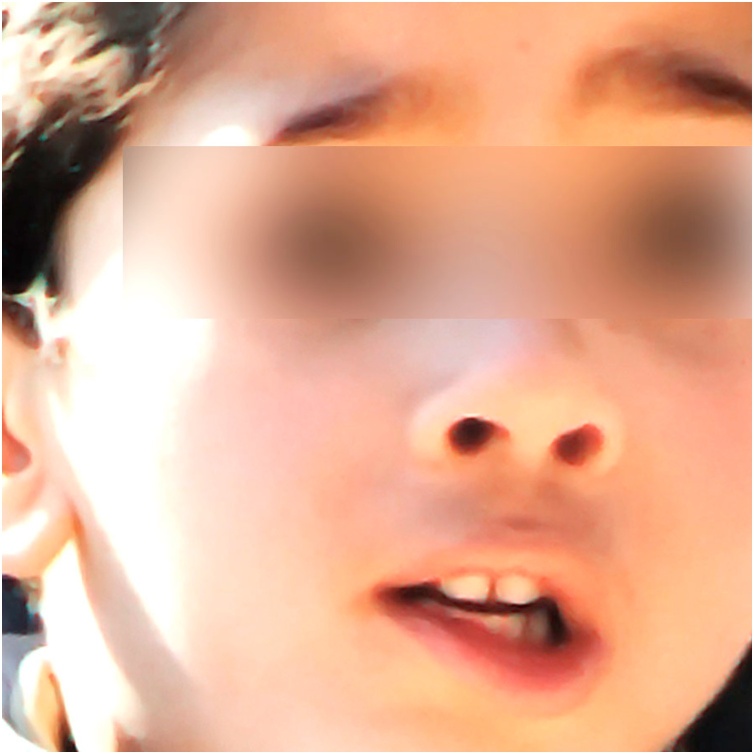
5 years old.

**Fig. 6:**
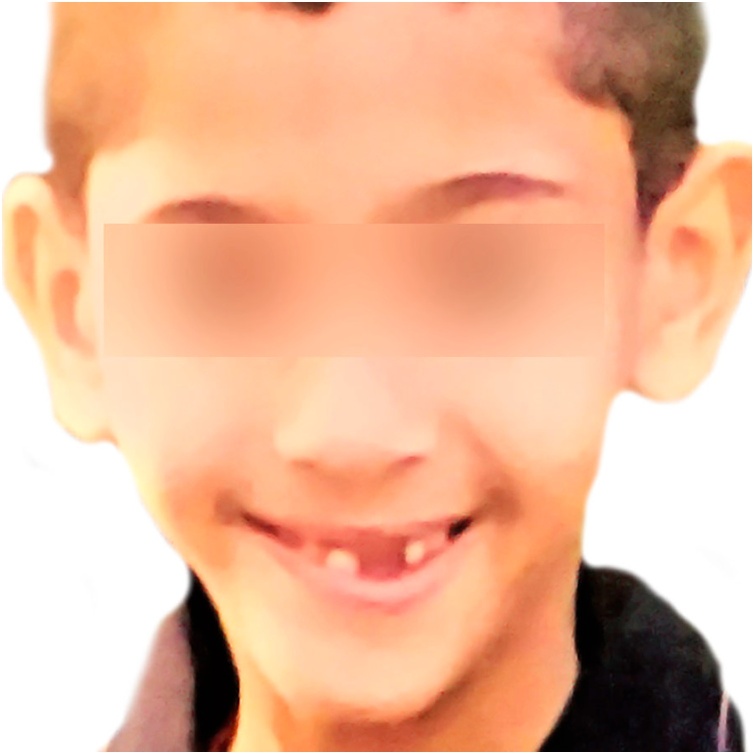
6 years old.

**Fig. 7:**
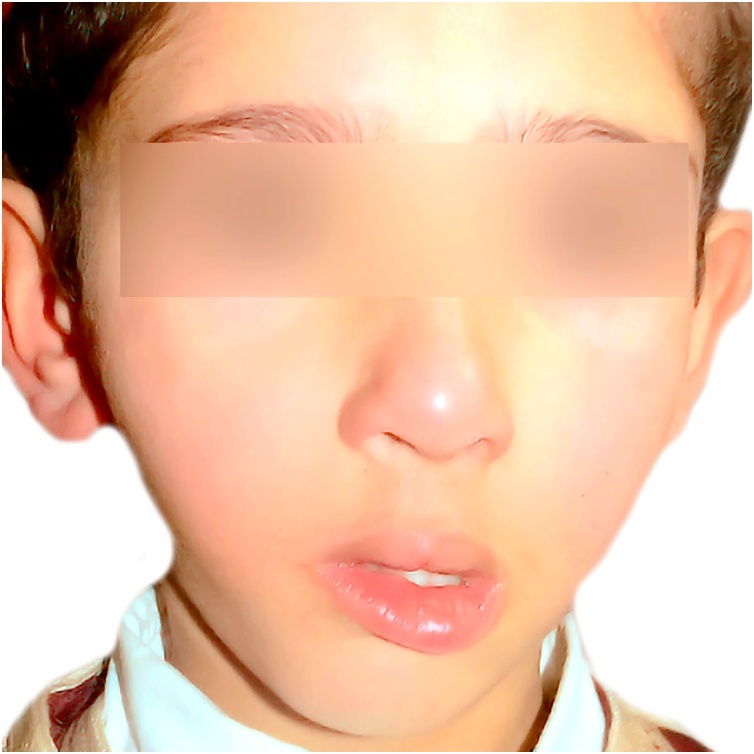
7 years old.

**Fig. 8:**
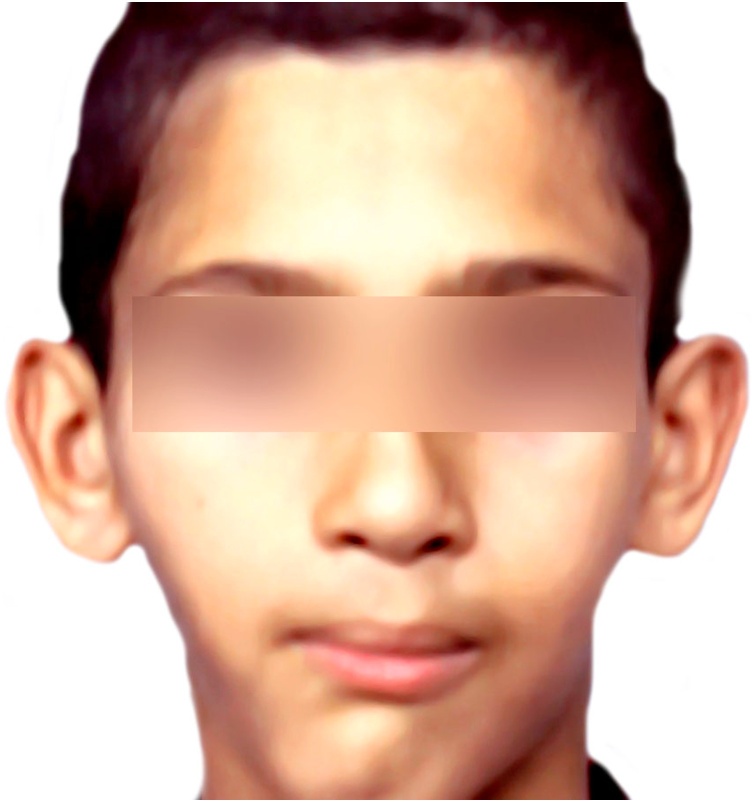
8 years old.

**Fig. 9:**
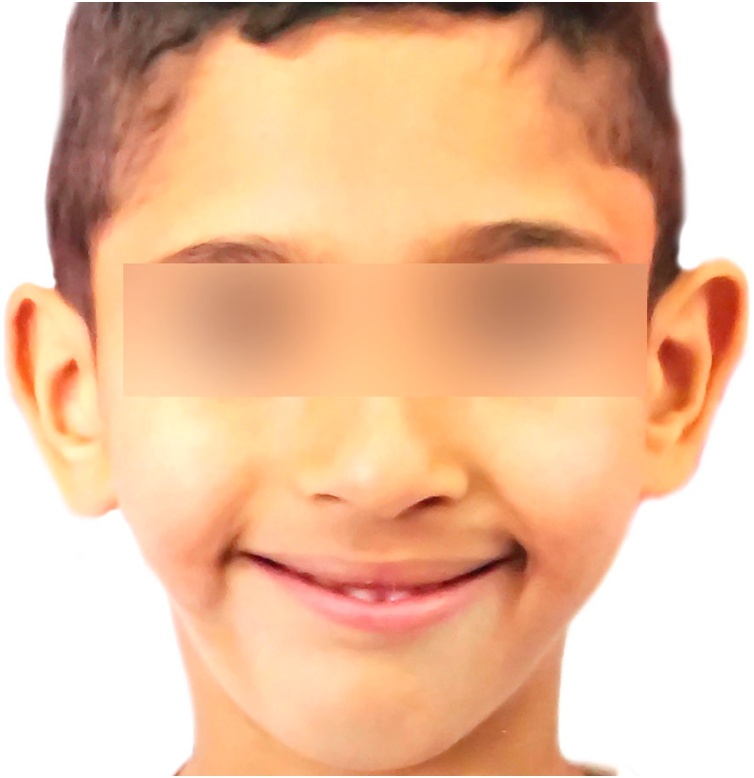
9 years old.

**Fig. 10:**
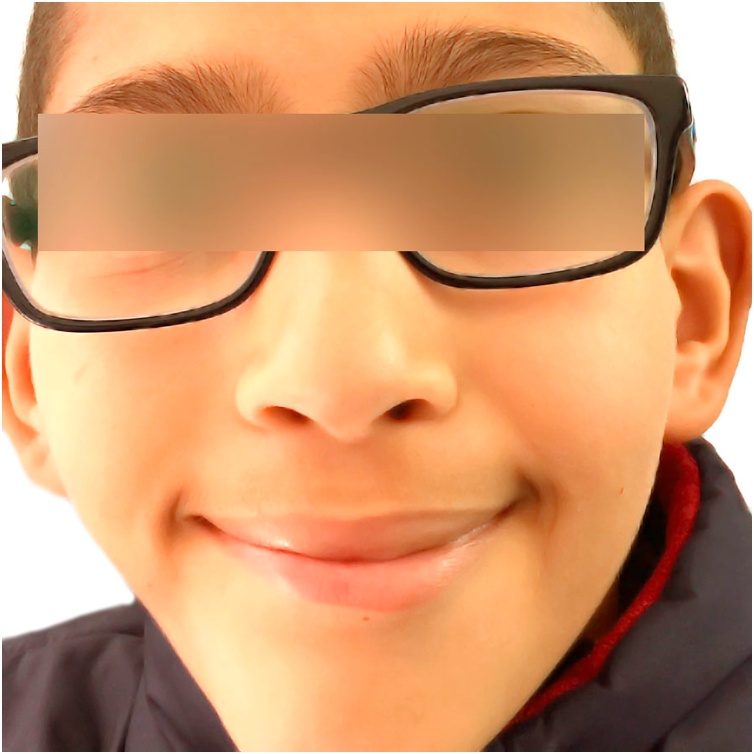
10 years old.

## 3 Discussion

Congenital hypoplasia of depressor angularis oris muscle (CHDAOM) is one of the rare causes of asymmetric crying facies in newborn. One of the first reports were published by Parmelee [5] who suggested a lesion of the facial nerve by an extreme intra-uterine flexion of the head with consecutive pressure. In fact, the pathogenesis of this condition is still a matter of debate. intrauterine molding was invoked as the cause of this syndrome by Hepner in 1951 [6]. The report of Papadatos et al. in 1974 also seemed to mention obstetric factors [7]. In another series of studies in 1969 Cayler postulated that the syndrome may be due to a subclinical viral infection occurring in the mother during the fifth week of pregnancy and to chromosomal breaks or deletions [8]. However, in our case report and other series [9], no noxious influences none obstetric factors during pregnancy were identified. It appears that the etiology of this syndrome is diverse and further studies are indicated to elucidate it

Congenital absence or hypoplasia of the depressor anguli oris muscle is recognizable since one corner of the mouth does not move downward and outward symmetrically with the other; asymmetry is particularly evident when the child is crying. Forehead wrinkling, eye closure, nasolabial fold depth, and tearing are symmetrical, and mentalis function is normal. Palpable thinning of the lateral portion of the lower lip is usually present on the affected side [1]. The symptoms improve with age through the decrease of crying frequency.

To understand the clinical features, knowledge of muscle anatomy and function is mandatory.

Muscles affected by this syndrome are: Musculus depressor labii inferioris arising from the anterior border of the mandible to the lower lip. Being innervated by the mandibular branch of the facial nerve, it pulls the lower lip downward and laterally, which allows active exposure of incisors of the mandible. The Depressor anguli oris arising from the lower border of the mandible and extends up to the modiolus creating the ability to draw the mouth downward and Mentalis musculus. The three muscles can be affected concomitantly. Isolated involvement of the Depressor anguli oris muscle has also been described. The mechanical dysfunction can be either linked to muscle innervation agenesis or to a defect thereof [10,11].

Asymmetric crying facies (ACF) in newborns has stimulated great interest because of its potential association with congenital anomalies but also in order to reassure families often worried by the situation.

Among congenital anomalies priority needs to be given to cardiac anomalies like atrial septal defect and patent ductus arteriosus. Mandibular hypoplasia and auricular dysplasia amongst head and neck anomalies. Skeletal, genitourinary, gastrointestinal central nervous system, and miscellaneous anomalies were also mentioned in literature [9].

The question which of the mimetic lower lip muscles is affected in CULLP is delicate. In response some studies used electromyographical investigations to confirm the myogenic nature of the syndrome which has shown: paucity of motor units, no fibrillation, no positive sharp waves, no high voltage motor units, and no prolongation of latency compared with the non-affected side. Also hypoplasia or congenital absence of the depressor anguli oris muscle have been assumed as the main reason for this mimic disorder [1,7,11]. Other investigators, however, pointed out that this condition may be due to a unilateral weakness of the depressor labii inferioris or the mentalis muscle [11].

Sonography as a non-invasive imaging tool of mimetic muscles was used in R- Roedel and al study showing the depressor anguli oris muscle, if present, as a dark band extending between the angle of the mouth and the mandibular border. In contrast, identification of the fibers of the depressor labii inferior muscle is difficult and not always successful: due to its course of fibers extending upward and forward, the muscle shows a lot of intrinsic echoes, which makes its separation from the surrounding connective tissue often difficult, even in cooperative infants authors concluded that ultrasonography may be a noninvasive tool yet non conclusive proof in this framework [11].

One study has been made to examine the anatomy of the mimetic muscles in patients with congenital lower lip palsy by biopsy reported by Levin et al., in which a difference in size of the mentalis muscle was described [11].

Differentiation between absence or hypoplasia of the facial musculature and facial nerve dysfunction are the main etiologies in this particular clinical presentation and often, a good anamnesis is sufficient to point in the right direction: it’s important to ask about birth trauma incidents ; the presence of familiar similar cases of asymmetric crying faces and keep in mind that some clinical presentations could guide through the diagnosis of hemihypertrophy - hemi atrophy syndromes like Goldenhar’s; hemifacial microsomia, Russel-silver dwarfism, Klippel-Trenaunay syndrome and Wilms’s association.

Treatment is difficult to plan. If patients or their families do not mind, no treatment is necessary. In some children, the cosmetic defect lessens with increasing age like for our young patient. It is not clear whether this is due to conscious avoidance of crying and grimacing, or to true improvement of muscle force.

The therapeutic armamentarium include surgical techniques : plastic-reconstructive procedures on the lower lip by the affected side ; Wedge-excision and fascia-lata sling or cheiloplasty; plication of the orbicularis oris muscle; transposition of the orbicularis muscle ; and digastric muscle transfer [11].

Weakening of the mimetic lower lip muscles on the non-affected side can be done using a selective neurectomy of the marginal mandibular branch of the opposite side [3] or throughout botulin-toxin infiltration [11].

As for our patient, among the large armamentarium of therapeutic options we opted for a wait and see strategy through photographic smile tracking leading to an evidence of Asymmetric crying faces over time improvement

## 4 Conclusion

The clinical hallmark of asymmetric crying facies (ACF) is a symmetric appearance of the oral aperture and lips at rest, but significant depression of one side of the lower lip with motion (crying or smiling). It can resolve spontaneously. This Condition’s challenge is triple:

- Drawing a distinction with a facial nerve palsy.
- Diagnosing the potential associated congenital anomalies specially the cardiac anomalies.
- Establishing a careful therapeutic approach including the psychosocial dimension.

Treatment with Botulin -toxin can offer temporary correction of ACF, with results lasting up to six months. An objective study is furthermore needed, the use of biometric measures is necessary to evaluate the symmetry of the commissure and its motion with measures at dynamic position.

## Declaration of Competing Interest

None.

## Sources of funding

None.

## Ethical approval

This type of study is exempt from ethnical approval in our institution.

## Consent

No identification characters.

## Author contribution

Sabrine Maamouri: Writing the paper.

Marouen Ben Rejeb: Lecturing and correction of the paper.

## Registration of research studies

N/A.

## Guarantor

Sabrine Maamouri.

## Provenance and peer review

Not commissioned, externally peer-reviewed.
